# Critérios otimizados de retorno ao esporte após o procedimento de Bristow-Latarjet para estabilização do ombro: Protocolo de revisão sistemática e metanálise

**DOI:** 10.1055/s-0046-1824729

**Published:** 2026-07-28

**Authors:** Ewerton Borges de Souza Lima, Rafaelly Stavale, Paulo Henrique Schmidt Lara, Benno Ejnisman, Arun J. Ramappa, Alberto de Castro Pochini

**Affiliations:** 1 Disciplina de Medicina Esportiva e Atividade Física, Departamento de Ortopedia e Traumatologia, Escola Paulista de Medicina, Universidade Federal de São Paulo, São Paulo, SP, Brasil; 2 Carl J. Shapiro Department of Orthopaedic Surgery, Beth Israel Deaconess Medical Center, Harvard Medical School, Boston, MA, Estados Unidos

**Keywords:** atletas, instabilidade articular, luxação do ombro, revisão sistemática como assunto, volta ao esporte, athletes, joint instability, return to sport, shoulder dislocation, systematic review as a topic

## Abstract

**Objetivo:**

Identificar e sintetizar os critérios de retorno ao esporte relatados após o procedimento de Bristow-Latarjet para instabilidade glenoumeral anterior em atletas, e avaliar o tempo associado e a taxa de retorno ao esporte e a recorrência de instabilidade, com o objetivo de informar uma combinação baseada em evidências de critérios de retorno ao esporte que possa otimizar o retorno antecipado, para minimizar a recorrência.

**Métodos:**

Este protocolo segue as diretrizes da declaração Preferred Reporting Items for Systematic review and Meta-Analysis-Protocols (PRISMA-P) e está registrado no International Prospective Register of Systematic Reviews (PROSPERO; CRD42024563618). Incluiremos estudos comparativos randomizados e não randomizados, estudos de coorte e de caso-controle, e séries de casos que relatem critérios explícitos de retorno ao esporte após Bristow-Latarjet em atletas, com seguimento mínimo de 12 meses. As buscas serão realizadas nas bases de dados PubMed, Web of Science, Embase, SciELO, CENTRAL e SPORTDiscus, além do Google Acadêmico para a literatura cinzenta, sem restrições de data, geografia ou idioma. Dois revisores farão a triagem e a extração de dados de forma independente, usando o programa Covidence (Veritas Health Innovation Ltd). Os resultados primários são o tempo até o retorno ao esporte, a taxa de retorno ao esporte e a recorrência (usando o ponto de tempo mais próximo de 12 meses, quando várias avaliações estão disponíveis). O risco de viés será avaliado utilizando a ferramenta Risk of Bias 2 (RoB 2; the Cochrane Collaboration) para ensaios clínicos randomizados (ECRs), a Newcastle-Ottawa Scale (NOS) para estudos comparativos não randomizados, e a Lista de Verificação de Avaliação Crítica para Séries de Casos do Joanna Briggs Institute (JBI) para séries de casos, e a certeza da evidência será classificada por meio da abordagem Grading of Recommendations Assessment, Development, and Evaluation (GRADE). Quando apropriado, metanálise de efeitos aleatórios e metarregressão exploratória serão realizadas; análises de subgrupo e de sensibilidade estão planejadas.

**Conclusão:**

Esta revisão sistemática fornecerá uma síntese centrada nos critérios das definições de retorno ao esporte e das estratégias de liberação após Bristow-Latarjet, relacionando cada critério a desfechos clinicamente relevantes (tempo até o retorno ao esporte, taxa de retorno ao esporte e recorrência). Espera-se que os resultados apoiem a tomada de decisão de retorno ao esporte de forma mais consistente e baseada em evidências, e orientem a futura validação prospectiva de combinações otimizadas de critérios de retorno ao esporte.

## Abstract (en)

**Objective:**

To identify and synthesize return-to-sport (RTS) criteria reported after the Bristow–Latarjet procedure for anterior glenohumeral instability in athletes, and to evaluate the associated time to RTS, RTS rate, and instability recurrence, with the aim of informing an evidence-based combination of RTS criteria that may optimize earlier RTS while minimizing recurrence.

**Methods:**

This protocol follows the guidelines of the Preferred Reporting Items for Systematic review and Meta-Analysis-Protocols (PRISMA-P) statement and it has been registered in the International Prospective Register of Systematic Reviews (PROSPERO; CRD42024563618). We will include randomized and nonrandomized comparative studies, cohort and case-control studies, and case series reporting explicit RTS criteria after Bristow–Latarjet in athletes, with a minimum follow-up of 12 months. Searches will be conducted in the PubMed, Web of Science, Embase, SciELO, CENTRAL and SPORTDiscus databases, as well as Google Scholar for grey literature, without restrictions on date, geography, or language. Two reviewers will independently screen and extract data using the Covidence (Veritas Health Innovation Ltd) software. The primary outcomes are time to RTS, RTS rate, and recurrence (using the time point closest to 12 months, when multiple assessments are available). The risk of bias will be assessed using the Risk of Bias 2 (RoB 2; the Cochrane Collaboration) for randomized clinical trials (RCTs), the Newcastle-Ottawa Scale (NOS) for nonrandomized comparative studies, and the Joanna Briggs Institute (JBI) Critical Appraisal Checklist for Case Series, and the certainty of the evidence will be rated according to the Grading of Recommendations Assessment, Development, and Evaluation (GRADE) approach. When appropriate, random-effects meta-analysis and exploratory meta-regression will be performed; subgroup and sensitivity analyses are planned.

**Conclusion:**

The systematic review will provide a criterion-centered synthesis of RTS definitions and clearance strategies after Bristow–Latarjet, linking each criterion to clinically-relevant outcomes (time to RTS, RTS rate, and recurrence). The findings are expected to support more consistent, evidence-informed RTS decision-making and guide future prospective validation of optimized RTS criteria combinations.

## Introdução

A luxação glenoumeral anterior é comum em indivíduos jovens e fisicamente ativos, 1 e pode evoluir para instabilidade do ombro e luxações recorrentes, 2 o que limita o desempenho esportivo e a qualidade de vida. 3 A recorrência é frequente em atletas de alto risco, o que torna a estabilização cirúrgica uma recomendação comum. 4

O procedimento de Bristow-Latarjet é uma técnica de bloqueio ósseo amplamente utilizada para tratar a instabilidade glenoumeral anterior (IGA), especialmente na presença de perda óssea glenóide, e envolve lesões de Hill-Sachs, falha na estabilização prévia dos tecidos moles ou em atletas com alto risco de recorrência, como aqueles que participam em esportes de contato ou esportes *overhead* (modalidades em que o atleta realiza movimentos repetitivos com os braços acima da linha da cabeça). 5 6 7

Ao restaurar o arco glenoidal anteroinferior e criar um efeito de tipoia dinâmico por meio do tendão do conjunto, o procedimento de Bristow-Latarjet fornece estabilidade confiável, com taxas de recorrência em torno de 2 a 5%, e a maioria dos atletas retorna ao esporte dentro de 3 a 6 meses. 5 6 7 8 No entanto, os resultados cirúrgicos variam entre os estudos, principalmente devido às diferenças nas características dos pacientes, nos protocolos de reabilitação e à falta de critérios padrão de retorno ao esporte para a liberação dos atletas à prática esportiva. 9

Essa heterogeneidade é evidente na literatura, com cronogramas de retorno ao esporte que variam de 6 semanas a 6 meses. Os protocolos que permitem a progressão mais precoce para a prática esportiva baseiam-se exclusivamente na fisiopatologia conhecida sobre o tempo de cicatrização do enxerto ósseo, com a liberação às vezes já considerada em 6 a 8 semanas, 10 ao passo que a abordagem mais conservadora atrasa o retorno ao esporte por aproximadamente 6 meses, com base no platô de melhora dos resultados funcionais. 6 8 11 Essa variação reflete a incerteza quanto ao que constitui “prontidão segura”, e limita o aconselhamento consistente aos atletas. 12

Até o momento, não há consenso universal quanto à prontidão para o retorno ao esporte após o procedimento de Bristow-Latarjet. Os critérios de retorno ao esporte publicados variam de liberação puramente baseada no tempo a abordagens multidomínio que incorporam exame clínico, amplitude de movimento, limiares de força, testes funcionais, confirmação de imagem e prontidão relatada pelo paciente. 13 Poucos estudos mapearam e compararam sistematicamente esses critérios, e ainda menos estudos tentaram propor combinações baseadas em evidências que equilibrem um retorno oportuno com baixo risco de recorrência. 13 14

Como resultado, a falta de critérios padrão de retorno ao esporte afeta a prática clínica, o que pode dificultar a otimização segura tanto do tempo até o retorno ao esporte quanto da estabilidade pós-operatória. Portanto, o objetivo deste artigo de protocolo é descrever a metodologia de uma revisão sistemática que identificará e sintetizará os critérios de retorno ao esporte relatados após o procedimento de Bristow-Latarjet e avaliará os resultados associados. O objetivo final da revisão é fornecer um critério baseado em evidências para apoiar o retorno antecipado ao esporte e minimizar a taxa de recorrência.

## Tipo de Estudo

Este é um protocolo para uma revisão sistemática que visa fornecer uma análise abrangente dos critérios de retorno ao esporte existentes após o procedimento de Bristow-Latarjet para IGA, identificar padrões e gerar hipóteses sobre combinações de critérios potencialmente úteis para um tempo otimizado de retorno ao esporte e menores taxas de recorrência. Seguirá as recomendações do *Cochrane Handbook for Systematic Reviews of Interventions* 15 e as diretrizes da declaração Preferred Reporting Items for Systematic Reviews and Meta-Analyses-Protocols (PRISMA-P) 16 ( Material Suplementar 1 ). Este estudo está registrado no International Prospective Register of Systematic Reviews (PROSPERO) sob o registro CRD42024563618.

## Pergunta da Revisão

A questão de pesquisa foi formulada utilizando a estratégia População, Intervenção, Comparação, Resultado ( *Outcome* ) e Tipo de Estudo ( *Study Type* ) (PICOS): (P) atletas com IGA submetidos ao procedimento de Bristow-Latarjet; (I) critérios de retorno ao esporte aplicados após o procedimento de Bristow-Latarjet; (C) diferentes critérios de retorno ao esporte; (O) tempo até o retorno ao esporte, a taxa de retorno ao esporte e recorrência de instabilidade/luxação; e (S) estudos clínicos que relatem critérios de retorno ao esporte após Bristow-Latarjet. Os critérios de inclusão e exclusão são detalhados na Tabela 1 .

**Tabela 1:** Critérios de inclusão e exclusão

Critério	Inclusão	Exclusão
**Tipo de estudo**	• Ensaios clínicos randomizados (ECRs); • Ensaios clínicos não randomizados; • Estudos de coorte prospectivos e retrospectivos; • Estudos de caso-controle; e • Série de casos (n ≥ 5 e com apresentação de dados quantitativos sobre os desfechos)	• Relatos de caso; • Notas técnicas, estudos em cadáveres e estudos biomecânicos; • Revisões sistemáticas, metanálises e outros tipos de revisão; e • Comentários, cartas para o editor, editoriais, opiniões de especialistas, resumos de conferências, pôsteres e capítulos de livros
**População**	• Atletas de todas as idades com instabilidade glenoumeral anterior (IGA) primária ou recorrente; e • Participantes submetidos ao procedimento de Bristow-Latarjet para o tratamento de IGA	• Pacientes com outras condições além de IGA (como instabilidades multidirecional e posterior); e • Pacientes que não são atletas ou cuja atividade física não está claramente definida
**Intervenção**	• Critérios de retorno ao esporte (tempo, força muscular, amplitude de movimento, dor, avaliação radiográfica etc.)	• Estudos que não definiram claramente os critérios de retorno ao esporte
**Desfechos**	• Tempo até o retorno ao esporte, taxa de retorno ao esporte, recorrência de lesões; e • Estudos com no mínimo 12 meses de seguimento	• Estudos que não definiram claramente o tempo até o retorno ao esporte, taxa de retorno ao esporte, recorrência de lesões; e • Estudos com menos de 12 meses de seguimento

Estudos que incluam pacientes que praticavam algum esporte antes da cirurgia serão elegíveis, independentemente do nível competitivo. Quando disponível, o perfil esportivo descrito em cada estudo será extraído e categorizado como recreativo, competitivo ou misto/não definido. As definições de retorno ao esporte também serão registradas conforme relatado pelos autores originais e categorizadas de acordo com o resultado avaliado, como retorno ao treinamento, à competição ou ao nível pré-lesão.

Estudos não randomizados serão incluídos, pois espera-se que as evidências disponíveis neste campo consistam predominantemente em estudos observacionais e séries de casos. Restringir a elegibilidade a estudos randomizados provavelmente excluiria uma proporção substancial das evidências relevantes sobre o retorno ao esporte e sobre os resultados de recorrência.

Esta revisão sistemática pretende responder às seguintes questões:

- Quais são os critérios de retorno ao esporte identificados na literatura para o procedimento de Bristow-Latarjet?
- Qual é o tempo até o retorno ao esporte, a taxa de retorno ao esporte e a taxa de recorrência para cada critério de retorno ao esporte?
- Qual é a melhor combinação de critérios de retorno ao esporte para a menor taxa de recorrência e o retorno antecipado ao esporte?

## Resultados

Os desfechos primários serão o tempo até o retorno ao esporte, a taxa de retorno ao esporte e a recorrência de instabilidade/luxação após o procedimento de Bristow-Latarjet. Para cada estudo incluído, extrairemos os critérios do retorno ao esporte e avaliaremos os respectivos desfechos.

Para melhorar a comparabilidade dos desfechos, aqueles relacionados à recorrência serão definidos *a priori* . A luxação confirmada será definida como um episódio documentado de luxação glenoumeral recorrente após o retorno ao esporte. A subluxação sintomática será definida como um episódio de instabilidade clinicamente relatado pelo paciente, sem luxação completa confirmada. A reoperação será definida como um procedimento cirúrgico subsequente realizado devido à instabilidade ou à falha recorrente após o procedimento-índice.

Os desfechos secundários incluirão retorno ao nível de esporte pré-lesão, medidas de resultado relatadas pelo paciente (Rowe, Western Ontario Shoulder Instability Index [WOSI], Single Assessment Numeric Evaluation [SANE], American Shoulder and Elbow Surgeons [ASES], ou outros escores validados), componentes funcionais objetivos incorporados nas definições de retorno ao esporte (amplitude de movimento, teste de força, testes de desempenho funcional) e complicações pós-operatórias (infecção, lesão neurovascular, complicações relacionadas ao enxerto, rigidez ou reoperação por razões que não sejam instabilidade recorrente). Também resumiremos como o retorno ao esporte foi definido e medido (retorno ao treinamento *versus* competição, considerações específicas do esporte).

Quando os desfechos são relatados em vários momentos do seguimento, extrairemos os dados da avaliação mais próxima de 12 meses de pós-operatório, desde que ocorra após esse período, para as análises primárias e secundárias. Os resultados avaliados antes de 12 meses não serão considerados nas análises comparativas.

## Métodos e Estratégia de Pesquisa

Uma pesquisa abrangente será realizada nas bases de dados PubMed, Embase, Web of Science, SciELO, CENTRAL e SPORTDiscus, com o Google Acadêmico utilizado para a literatura cinzenta. PubMed e Embase foram incluídas por sua ampla indexação biomédica; Web of Science, por sua cobertura multidisciplinar; SciELO, por sua literatura regional (revistas latino-americanas e ibéricas); CENTRAL, por estudos controlados; e SPORTDiscus, por literatura de medicina esportiva e reabilitação relevante para resultados de retorno ao esporte.

A estratégia de busca ( Tabela 2 ) foi intencionalmente centrada nos termos presentes no título/resumo para melhorar a especificidade e identificar estudos com ênfase clara no tema da revisão. Os termos selecionados incorporam conceitos relevantes de Medical Subject Headings (MeSH)/Embase Subject Headings (Emtree) e terminologia padronizada comumente utilizada na literatura. Esses termos foram testados iterativamente durante o desenvolvimento da estratégia, e essa abordagem também facilita maior consistência entre as diferentes bases de dados pesquisadas, melhorando, assim, a reprodutibilidade da estratégia de busca.

**Tabela 2:** Estratégias de busca para cada base de dados

Base de dados	Estratégias de busca
**PubMed**	(shoulder instability[Title/Abstract] OR glenohumeral instability[Title/Abstract] OR glenohumeral dislocation[Title/Abstract] OR anterior glenohumeral instability[Title/Abstract] OR anterior shoulder instability[Title/Abstract]) AND (Latarjet[Title/Abstract] OR Bristow[Title/Abstract]) AND (return time[Title/Abstract] OR return sport*[Title/Abstract] OR return play[Title/Abstract] OR return practice[Title/Abstract]) AND (recurrence[Title/Abstract] OR recurrent[Title/Abstract] OR re-dislocation[Title/Abstract] OR re-injury[Title/Abstract] OR instability[Title/Abstract] OR apprehension[Title/Abstract] OR fail[Title/Abstract])
**Web of Science**	(shoulder instability OR glenohumeral instability OR glenohumeral dislocation OR anterior glenohumeral instability OR anterior shoulder instability) AND (Latarjet OR Bristow) AND (return time OR return sport* OR return play OR return practice) AND (recurrence OR recurrent OR re-dislocation OR re-injury OR instability OR apprehension OR fail)
**SciELO**	(“shoulder instability” OR “glenohumeral instability” OR “glenohumeral dislocation” OR “anterior glenohumeral instability” OR “anterior shoulder instability” OR “instabilidade do ombro” OR “instabilidade glenoumeral” OR “luxação glenoumeral” OR “instabilidade anterior do ombro” OR “instabilidade glenoumeral anterior” OR “inestabilidad de hombro” OR “inestabilidad glenohumeral” OR “luxación glenohumeral” OR “inestabilidad anterior de hombro” OR “inestabilidad glenohumeral anterior”) AND (Latarjet OR Bristow) AND (“return time” OR “return sport*” OR “return to sport*” OR “return to play” OR “return to practice” OR “retorno ao esporte” OR “retorno à prática esportiva” OR “retorno ao jogo” OR “tempo de retorno” OR “retorno al deporte” OR “retorno a la práctica deportiva” OR “regreso al deporte” OR “tiempo de retorno”) AND (recurrence OR recurrent OR “re-dislocation” OR reinjury OR “re-injury” OR instability OR apprehension OR failure OR recidiva OR recorrência OR reluxação OR “nova luxação” OR instabilidade OR “sinal de apreensão” OR falha OR recurrencia OR recidiva OR “re-luxación” OR “nueva luxación” OR inestabilidad OR “signo de aprensión” OR fallo)
**Embase**	(“shoulder instability”:ti,ab OR “glenohumeral instability”:ti,ab OR “glenohumeral dislocation”:ti,ab OR “anterior glenohumeral instability”:ti,ab OR “anterior shoulder instability”:ti,ab) AND (latarjet:ti,ab OR bristow:ti,ab) AND (“return time”:ti,ab OR “return to sport*”:ti,ab OR “return to play”:ti,ab OR “return to practice”:ti,ab) AND (recurrence:ti,ab OR recurrent:ti,ab OR “re-dislocation”:ti,ab OR “re-injury”:ti,ab OR instability:ti,ab OR apprehension:ti,ab OR fail*:ti,ab)
**CENTRAL**	((“shoulder instability” OR “glenohumeral instability” OR “glenohumeral dislocation” OR “anterior glenohumeral instability” OR “anterior shoulder instability”):ti,ab,kw) AND ((Latarjet OR Bristow):ti,ab,kw) AND ((“return time” OR “return sport*” OR “return play” OR “return practice”):ti,ab,kw) AND ((recurrence OR recurrent OR “re-dislocation” OR “re-injury” OR instability OR apprehension OR fail):ti,ab,kw)
**SPORTDiscus**	(TI (“shoulder instability” OR “glenohumeral instability” OR “glenohumeral dislocation” OR “anterior glenohumeral instability” OR “anterior shoulder instability”) OR AB (“shoulder instability” OR “glenohumeral instability” OR “glenohumeral dislocation” OR “anterior glenohumeral instability” OR “anterior shoulder instability”)) AND (TI (Latarjet OR Bristow) OR AB (Latarjet OR Bristow)) AND (TI (“return time” OR “return sport*” OR “return play” OR “return practice”) OR AB (“return time” OR “return sport*” OR “return play” OR “return practice”)) AND (TI (recurrence OR recurrent OR “re-dislocation” OR “re-injury” OR instability OR apprehension OR fail) OR AB (recurrence OR recurrent OR “re-dislocation” OR “re-injury” OR instability OR apprehension OR fail))
**Google Scholar**	(intitle:Latarjet OR intitle:Bristow) (“shoulder instability” OR “glenohumeral instability” OR “glenohumeral dislocation” OR “anterior glenohumeral instability” OR “anterior shoulder instability”) (“return to sport” OR “return to sports” OR “return to play” OR “return to practice” OR “return time”) (recurrence OR recurrent OR “re-dislocation” OR “reinjury” OR “re-injury” OR instability OR apprehension OR failure)

Os termos de pesquisa abrangerão conceitos relacionados à instabilidade ou luxação anterior do ombro/glenoumeral, ao procedimento de Bristow-Latarjet, ao retorno ao esporte e aos resultados de recorrência/instabilidade. Não haverá restrições de data, geografia ou idioma. Se necessário, uma ferramenta de tradução será utilizada na coleta de dados. Uma pesquisa atualizada será realizada ao final do estudo para garantir a inclusão de quaisquer estudos recentes.

Dois revisores independentes farão a triagem dos títulos e resumos dos artigos identificados quanto à elegibilidade inicial. Artigos duplicados serão removidos com o software Covidence (Veritas Health Innovation Ltd). Artigos com texto completo de estudos potencialmente relevantes serão avaliados para determinar a elegibilidade com base nos critérios de inclusão e exclusão. As divergências serão resolvidas por um terceiro revisor sênior.

Para evitar a dupla contagem de participantes, as populações de estudo potencialmente sobrepostas serão avaliadas por meio da comparação do centro de estudo, do período de recrutamento, do grupo de autores e das características da amostra. Quando houver suspeita de sobreposição, o conjunto de dados mais abrangente ou mais informativo será incluído, e coortes duplicadas ou substancialmente sobrepostas serão excluídas.

Os motivos das exclusões serão documentados em uma tabela na avaliação final. Nos resultados, relataremos o processo de seleção do estudo, utilizando o diagrama de fluxo da PRISMA.

## Extração de Dados

Conforme já dito, dois revisores extrairão dados de forma independente usando o programa Covidence. As seguintes informações serão coletadas de cada estudo incluído: dados de identificação do estudo, características da amostra, detalhes da intervenção, medidas de desfecho, dados sobre retorno ao esporte, recorrência e complicações, risco de viés e qualidade do estudo. Os dados serão inseridos em um formulário de extração padronizado ( Tabela 3 ) no Covidence e, posteriormente, exportados para o Excel (Microsoft Corp.) para gerenciamento e análise de dados.

**Tabela 3:** Tabela de extração de dados com explicação de cada item

Categoria	Detalhes
**Identificação dos estudos**	
Identificação do estudo	Identificador único
Autores	Todos os autores
Título	Título completo da publicação
Ano de publicação	Ano de publicação
Nome do periódico	Nome do periódico
**Detalhes dos participantes**	
Idade	Idade média e variação de idade dos participantes
Sexo	Número ou porcentagem dos participantes do sexo feminino e masculino
Tamanho da amostra	Número total dos participantes
Critério de inclusão	Critérios específicos usados para inclusão
Critério de exclusão	Critérios específicos usados para exclusão
Características basais	Detalhes das características basais (como esporte, lesões anteriores, nível de competição, duração dos sintomas)
**Detalhes da intervenção**	
Técnica cirúrgica	Bristow e/ou Latarjet, aberta ou artroscópica, parafusos ou botão de sutura
Protocolo de reabilitação	Informações do protocolo de reabilitação do pós-operatório
**Medidas de desfecho**	
Duração do seguimento	Tempo médio do seguimento
Momento de avaliação da medida de desfecho	Momento do seguimento em que os desfechos foram medidos
Escores funcionais	Detalhes dos escores funcionais (como Constant-Murley, Western Ontario Shoulder Instability Index [WOSI], e American Shoulder and Elbow Surgeons [ASES])
**Data de retorno ao esporte**	
Definição do retorno ao esporte	Como o retorno ao esporte é definido (participação total, apenas em treinamento)
Tempo até o retorno ao esporte	Tempo médio até o retorno ao esporte
Nível do retorno	Se os atletas retornaram para o mesmo nível de competição ou para um nível diferente
Taxa de retorno ao esporte	Taxa de participantes que conseguiram retornar ao esporte
**Recorrências e complicações**	
Taxa de recorrência	Dados detalhados referentes à taxa de recorrência e definição (o prazo final é 12 meses)
Complicações	Quaisquer complicações relatadas no pós-operatório (infecção, falha do enxerto)
**Risco de viés e avaliação da qualidade**	
Risco de viés	Resultados do risco de viés das ferramentas (Risk of Bias 2 [ROB2] para ensaios clínicos randomizados, Newcastle-Ottawa Scale [NOS] para ensaios clínicos não-randomizados e a Lista de Verificação de Avaliação Crítica para Séries de Casos do Joanna Briggs Institute [JBI])
Escore de qualidade	Escore ou classificação geral da qualidade de cada estudo
Nível de evidência	Níveis de evidência do Oxford Centre for Evidence-Based Medicine (OCEBM)

## Avaliação de Risco de Viés

Dois revisores avaliarão, de forma independente, o risco de viés de todos os estudos incluídos, e fornecerão uma justificativa por escrito para cada julgamento; as divergências serão resolvidas por consenso ou por consulta a um terceiro revisor sênior. Ensaios clínicos randomizados (ECRs) serão avaliados com a ferramenta Risk of Bias 2 (RoB 2; the Cochrane Collaboration); estudos comparativos não randomizados serão avaliados com Newcastle-Ottawa Scale (NOS); e séries de casos serão avaliadas com a Lista de Verificação de Avaliação Crítica para Séries de Casos do Joanna Briggs Institute (JBI).

O viés de publicação será avaliado por meio de gráficos de funil e do teste de Egger somente quando mais de dez estudos estiverem disponíveis para uma determinada metanálise, pois esses métodos não são considerados informativos quando o número de estudos incluídos é pequeno.

## Qualidade da Evidência

A qualidade geral da evidência para cada desfecho primário será avaliada com base na abordagem Grading of Recommendations Assessment, Development and Evaluation (GRADE). 17 A qualidade das evidências será classificada como alta, moderada, baixa ou muito baixa, com base nos domínios de risco de viés, inconsistência, indireta, imprecisão e viés de publicação. As evidências randomizadas começarão com alta certeza e podem ser classificadas para baixo nesses domínios, ao passo que as evidências não randomizadas começarão com baixa certeza e podem ser classificadas para baixo ou, quando apropriado, para cima, de acordo com a orientação do GRADE. Apresentaremos os julgamentos do GRADE e os principais resultados em uma tabela (“Resumo dos Resultados”) dos desfechos primários.

## Dados Não Encontrados

Quando os dados dos desfechos não estiverem disponíveis, forem imprecisos ou forem relatados de forma incompleta, tentaremos obter as informações necessárias entrando em contato com os autores correspondentes. Se os dados permanecerem indisponíveis, relataremos a extensão e a natureza da indisponibilidade e incluiremos o estudo na síntese descritiva, excluindo-o de qualquer análise quantitativa que exija as informações ausentes. Quando possível, as medidas de dispersão ausentes serão estimadas a partir de outras estatísticas disponíveis, utilizando métodos padrão. Não serão incluídos valores de resultados ausentes no nível do participante; em vez disso, as análises serão baseadas nos dados disponíveis, e o impacto potencial dos dados ausentes será explorado por meio de análises de sensibilidade, quando apropriado.

## Análise de Subgrupos

Análises de subgrupos serão realizadas quando dados suficientes estiverem disponíveis para explorar fontes potenciais de heterogeneidade e modificadores de efeito clinicamente relevantes. Os subgrupos planejados incluem: tipo de critério de retorno ao esporte, definição de retorno ao esporte, características esportivas (colisão/contato *versus* não contato; overhead versus não-overhead), nível competitivo (recreativo *versus* competitivo) e técnica cirúrgica (aberta *versus* artroscópica). Quando possível, os efeitos dos subgrupos serão avaliados comparando estimativas agrupadas entre subgrupos e, se apropriado, por meio de metarregressão; os resultados dos subgrupos serão interpretados com cautela e considerados exploratórios.

## Análise de Sensibilidade

Análises de sensibilidade serão conduzidas para avaliar a robustez dos resultados e o impacto das principais decisões metodológicas. Quando dados suficientes estiverem disponíveis, repetiremos as metanálises após a exclusão de estudos com alto risco de viés.

## Análise Estatística

Todas as análises estatísticas serão baseadas em dados agregados relatados nos estudos incluídos. O gerenciamento de dados e as análises descritivas serão realizados com os programas Covidence e Excel. Metanálises e metarregressões serão realizadas com um programa estatístico, como Stata (StataCorp LLC) e/ou R (R Foundation for Statistical Computing). A significância estatística será definida como valores de *p* bilaterais < 0,05 para a maioria das análises, exceto quando especificado de outra forma ( *p* < 0,10 para testes de heterogeneidade). Todas as estimativas serão apresentadas com ICs95%. A concordância entre os avaliadores na seleção do estudo e nas avaliações de risco de viés será quantificada por meio da estatística Kappa de Cohen, com IC95%. Para resultados contínuos, extrairemos as médias e os desvios padrão. Para desfechos dicotômicos, calcularemos proporções com ICs95% para cada estudo ou grupo de estudo.

**Fig. 1:**
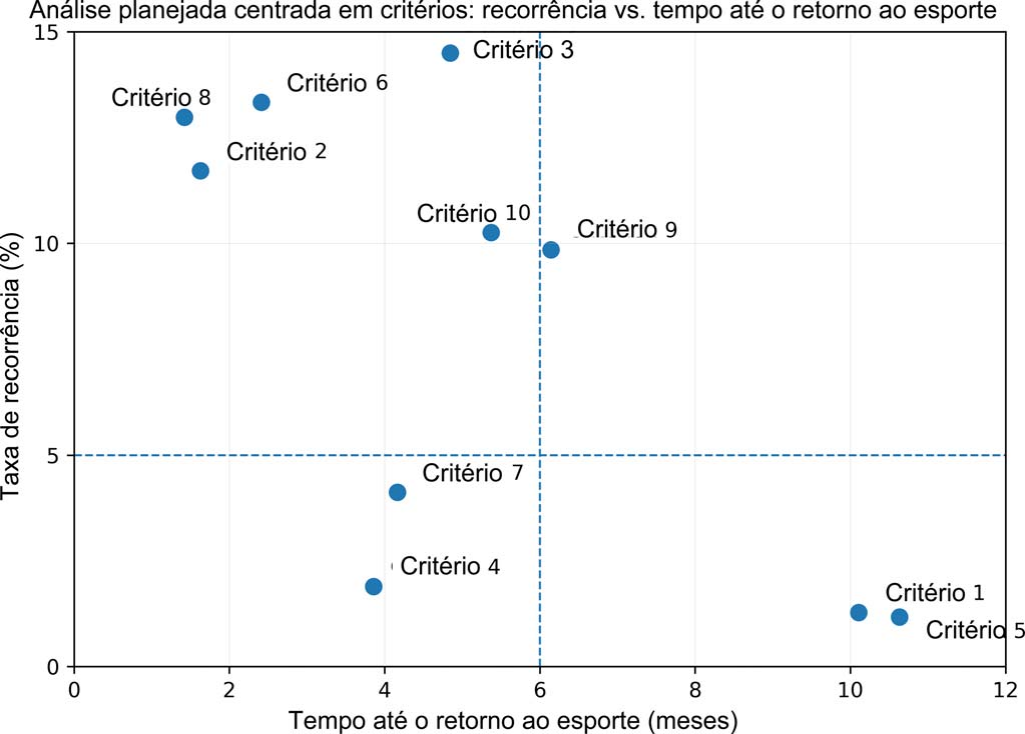
Exemplo conceitual da apresentação de resultados planejada, centrada em critérios. Cada ponto representa um critério de retorno ao esporte (critérios 1–10), plotado em função do tempo até o retorno ao esporte (meses, eixo x) e da taxa de recorrência (porcentagem, eixo y). Pontos mais próximos do canto inferior esquerdo indicam retorno antecipado ao esporte e menor recorrência. Esta figura é apenas ilustrativa; não representa resultados preliminares.

Inicialmente, será realizada uma síntese narrativa para descrever: os critérios de retorno ao esporte utilizados em cada estudo, as características das populações, os detalhes cirúrgicos, os protocolos de reabilitação, o tempo de seguimento e o risco de viés. As tabelas de resumo apresentarão, para cada estudo incluído, os critérios de retorno ao esporte, o tempo até o retorno ao esporte, a taxa de retorno ao esporte e a taxa de recorrência. Além disso, desenvolveremos um gráfico de dispersão que exiba a relação entre a taxa de recorrência e o tempo de retorno ao esporte para cada critério, para permitir uma comparação visual mais clara e apoiar a seleção dos critérios clinicamente mais úteis para a prática ( Fig. 1 ).

Dado que se espera que muitos estudos elegíveis sejam séries de casos ou outros projetos de braço único, os dados de braço único serão incluídos na síntese quantitativa quando apropriado. Para resultados de proporção, como taxas de retorno ao esporte e de recorrência, as estimativas agrupadas serão calculadas por meio de uma metanálise de efeitos aleatórios de proporção, com uma transformação de estabilização de variância apropriada. Estudos com eventos raros ou dados de evento zero não serão excluídos somente com base nisso: serão aplicados métodos estatísticos adequados.

O agrupamento quantitativo de tempo até o retorno ao esporte será realizado somente quando os estudos relatarem dados suficientemente homogêneos em formatos compatíveis. Quando esses resultados são relatados como medianas com intervalos interquartis (IIQ) ou intervalos, serão resumidos descritivamente e convertidos para agrupamento apenas quando metodologicamente apropriado.

A metanálise será realizada quando os estudos forem considerados suficientemente homogêneos quanto à população, à intervenção e às definições de resultados. Serão utilizados modelos de efeitos aleatórios tendo em vista a heterogeneidade clínica e metodológica esperada. A heterogeneidade estatística será avaliada pelo teste Q de Cochran e quantificada pela estatística I ^2^ , interpretada como baixa (∼ 25%), moderada (∼ 50%) ou alta (∼ 75%).

Para explorar se diferentes combinações de critérios de retorno ao esporte estão associadas à variação do tempo até o retorno ao esporte e às taxas de recorrência, podem ser realizadas análises de metarregressão em nível de estudo. O preditor primário será a categoria de critérios de retorno ao esporte utilizada em cada estudo, ao passo que covariáveis adicionais, incluindo tipo de esporte, nível de competição, abordagem cirúrgica, duração do seguimento e risco geral de viés, podem ser consideradas, dependendo da disponibilidade de dados. Qualquer metarregressão será considerada exploratória e conduzida apenas se possível, com pelo menos dez estudos por covariável, como regra geral, para reduzir o risco de sobreajuste e de achados espúrios.

## Discussão

O retorno ao esporte após o procedimento de Bristow-Latarjet é frequentemente apresentado como uma das principais vantagens dessa técnica, 18 19 mas os critérios usados para liberar os atletas permanecem altamente variáveis. Essa falta de padronização pode contribuir tanto para o retorno prematuro ao esporte, com risco potencial de recorrência, quanto para atrasos excessivamente conservadores que prolongam o tempo longe do esporte. 19

Pesquisadores e cirurgiões ortopédicos atualmente usam uma ampla gama de abordagens para permitir o retorno ao esporte, incluindo liberação baseada no tempo, marcos clínicos (dor, estabilidade, amplitude de movimento), testes objetivos de força, medidas de desempenho funcional, confirmação por imagem da posição e da cicatrização do enxerto e prontidão relatada pelo paciente. 13 14 Esses critérios podem ser aplicados isoladamente ou combinados de diferentes maneiras. No entanto, mesmo quando domínios semelhantes são considerados, os limiares raramente são padronizados, e o próprio retorno ao esporte é definido de forma inconsistente, como, por exemplo, retorno ao treinamento *versus* retorno à competição, ou retorno a qualquer nível *versus* retorno ao nível pré-lesão. 9 Essa variabilidade cria problemas importantes de medição, pois os atletas podem ser classificados como “retornados” segundo padrões marcadamente diferentes, e a recorrência pode ser relatada com definições não uniformes.

A falta de critérios padrão de retorno ao esporte limita a comparabilidade entre os estudos, aumenta o risco de inferências tendenciosas e desafia a tomada de decisões baseadas em evidências clínicas. No entanto, uma revisão sistemática é adequada para abordar esse problema, pois permite um mapeamento estruturado de como os critérios de retorno ao esporte são definidos na literatura e de como se relacionam com os resultados em ambientes heterogêneos. Nosso protocolo inclui uma metodologia rigorosa, que fortalece a validade interna, mas prevemos desafios práticos: relatórios incompletos sobre as definições de retorno ao esporte, variabilidade nos cronogramas de seguimento, terminologia inconsistente de resultados e disponibilidade limitada de medidas de efeito comparáveis entre os estudos. Como resultado, a síntese quantitativa só pode ser viável para resultados e subconjuntos selecionados, e a síntese narrativa permanecerá essencial para preservar o significado clínico.

Os desfechos primários desta revisão foram escolhidos porque refletem o principal equilíbrio clínico após a estabilização: permitir um retorno mais precoce, mantendo a estabilidade duradoura. Ao vincular cada critério de retorno ao esporte a esses resultados, a revisão visa a sustentar uma justificativa clínica mais explícita na seleção de estratégias de liberação. Esperamos descobrir que os critérios baseados no tempo, por si só, são comuns e podem estar associados a uma ampla variabilidade no tempo de retorno ao esporte e na recorrência, ao passo que as abordagens de múltiplos domínios podem oferecer um perfil de segurança mais consistente. Uma dificuldade fundamental será separar o efeito do próprio critério de retorno ao esporte de fatores de confusão, como o tipo de esporte, o nível competitivo, a técnica cirúrgica, a intensidade de reabilitação e a perda óssea basal, todos os quais podem influenciar as decisões e os resultados de liberação.

Algumas limitações são prováveis. A base de evidências pode ser dominada por desenhos observacionais e séries de casos, com risco variável de viés e de relato incompleto, o que pode reduzir a certeza e restringir a metanálise. Diferenças nas definições dos desfechos, nos pontos de tempo de seguimento e no tratamento de eventos recorrentes podem limitar ainda mais o agrupamento e exigir estratificação cuidadosa e ênfase na síntese narrativa. Apesar desses desafios, a força deste estudo reside em sua abordagem centrada em critérios e em seu foco em resultados clinicamente aplicáveis. Ao sintetizar os critérios de retorno ao esporte e seus resultados associados, e ao classificar a certeza da evidência, esta revisão pode ajudar a mover as decisões de retorno ao esporte da tradição para padrões mais claros e informados por evidências, informar a tomada de decisão compartilhada com atletas e orientar futuros estudos prospectivos que testem caminhos de retorno ao esporte padronizados e baseados em critérios após Bristow-Latarjet.
